# On the role of composition and processing parameters on the microstructure evolution of Ti-xMo alloys

**DOI:** 10.1186/s13065-019-0529-3

**Published:** 2019-01-29

**Authors:** Michael. Y. Mendoza, Peyman Samimi, David. A. Brice, Iman Ghamarian, Matt Rolchigo, Richard LeSar, Peter. C. Collins

**Affiliations:** 1 Department of Materials Science and Engineering, Iowa State University, Ames, IA 50011 USA; 2 Center for Advanced Non-Ferrous Structural Alloys (CANFSA), Ames, USA; 3 Department of Materials Science and Engineering, Texas A&M University, College Station, TX 77840 USA; 4 School of Materials Engineering, Purdue University, West Lafayette, IN 47907 USA; 5 Department of Materials Science and Engineering, University of Michigan, Ann Arbor, MI 48109 USA

**Keywords:** Titanium alloys, Electron microscopy, Grain refinement, Additive manufacturing, Energy density, Constitutional supercooling

## Abstract

Laser Engineered Net Shaping (LENS™) was used to produce a compositionally graded Ti-xMo (0 ≤ x ≤ 12 wt %) specimen and nine Ti-15Mo (fixed composition) specimens at different energy densities to understand the composition–processing–microstructure relationships operating using additive manufacturing. The gradient was used to evaluate the effect of composition on the prior-beta grain size. The specimens deposited using different energy densities were used to assess the processing parameters influence the microstructure evolutions. The gradient specimen did not show beta grain size reduction with the Mo content. The analysis from the perspective of the two grain refinement mechanisms based on a model known as the Easton & St. John, which was originally developed for aluminum and magnesium alloys shows the lower bound in prior-beta grain refinement with the Ti–Mo system. The low growth restriction factor for the Ti-Mo system of *Q *= 6,5C_0_ explains the unsuccessful refinement from the solute-based mechanism. The energy density and the grain size are proportional according to the results of the nine fixed composition specimens at different energy densities. More energy absorption from the material represents bigger molten pools, which in turn relates to lower cooling rates.

## Introduction

In the recent years, several studies have been conducted to assess the influence of various alloys [1, 2] and additive manufacturing processing parameters [3, 4] on the microstructure evolution of titanium alloys. Knowing the microstructure is determinant to predict the properties of the material and for this reason, the effects of composition and processing parameters are critical for the development of new alloys. The titanium market has been traditionally dominated by the aerospace industry, but recently other industries as biomedical and chemical have seen increasing demands for titanium and its alloys [5]. The continuous development of new additive manufacturing techniques and the growing applicability of titanium alloys demand the understanding of the influence of composition and processing on different aspects of microstructure such as grain size, grain orientation (texture), compositional fluctuations, porosity and any change in the morphology.

Additive manufacturing (AM) is associated with conditions that lie far away from the thermodynamic equilibrium, leading to lower solute partitioning compared to casting due to the rapid solidification nature of the process and the possibility to affect a range of compositions in a single specimen by adopting a combinatorial approach. AM is fundamentally a combination of physical phenomena including heat transfer, fluid dynamics, phase transformations and thermophysical properties that define the microstructure evolutions [6]. There are several efforts to simulate the molten pool [7, 8] and the solidification process [9] in AM to understand and predict the microstructure.

Most of the previous work has focused on the processing variation as a function of distance from the build plate [10–13], part size [11] and the energy input and traverse speed [3, 13–15]. However, in the main, the vast majority of previous work has focused on the alloy Ti-6Al-4V.

From the chemical composition perspective, several studies regarding the grain refinement of Ti-based alloys with different alloy elements have shown the applicability of two refinement mechanisms by the Easton & St. John model [16–20], but not generally for additive manufacturing. For solidification, the solute-based mechanism considers the grain growth restriction factor and the nuclei-based mechanism takes into account the availability of nucleant particles. However, those studies were using conventional casting processes. Combinatorial approaches using additive manufacturing techniques have been performed on different Ti-based alloys. Microstructure evolutions as a function of composition gradient in the as-deposited condition from elemental blends for several binary alloys such as Ti-xCr [21], Ti-xAl [22] and Ti-xTa [23] have been studied in the past. In the specific case of Ti-Mo system, our previous work has been focused on the composition–microstructure relationship. The Ti-Mo system has been considered as an important material due to its potential corrosion resistance, ductility and biocompatibility depending on its Mo content and the associated metallurgical condition [24]. Studies by Furuhara et al. [25] on alloy buttons of Ti-Mo in the range of 10 to 40 wt% indicate that at certain aging temperatures the lpha laths form on the beta grain boundaries, and grow into the beta grains. Ho et al. reported the crystal structure and the morphology variations on cast Ti-Mo alloys with molybdenum content from 6 to 20 wt% [26] and more recently Zhao et al. focused on the biomedical applications as vertebral fixation using a Mo content of 15–18 wt% [27]. However, these studies were not specifically focused on the additive manufacturing processes and the as-deposited condition. One published work was focused on a compositional gradient, from elemental Ti to Ti-40 wt% alloy. It was deposited using the Laser Engineered Net-Shaping (LENS™) process by Collins et al. [28]. The microstructure across the gradient was α/β with the reduction of α fraction inasmuch the Mo content increases. In addition, microhardness and α lath thickness was quantified, observing a reduction from 1.04 μm at ~ 1.6 wt% Mo to 0.31 μm (310 nm) at ~ 18 wt% Mo [28]. Nevertheless, an assessment of the prior-beta grain refinement using the Easton & St. John model concepts was not performed. The evaluation of Mo as a grain refiner and the extended applicability of the model to the Ti-Mo system are important for a better understanding of the composition-microstructure relationship in Ti-alloys.

In this work, the purpose is to observe the changes in the microstructure features associated with the variations of composition and processing parameters of the additive manufacturing of the LENS™ process. In our previous research work [29], we were focus on the Ti–W system due to its high growth restriction factor of about 22.65C_0_. Here our interest is to evaluate the lower bound in terms of grain refinement from the perspective of the growth restriction factor that for the Ti-Mo system is 6.5C_0_. For this purpose, a compositional gradient of Ti-xMo (0 ≤ x ≤ 12 wt%) and nine specimens with different energy densities within the range of ~ 149 to ~ 448 kJ cm^−3^ for a fixed composition of Ti-15Mo were produced using LENS™ technology. Here, prior beta grain refinement through the concept of refinement mechanisms was the primary focus, although clearly grain refinement would lead to other potential benefits such as texture modification [30] and potential impacts on the mechanical properties. In addition to prior-beta grain size, other characteristics such as porosity and unmelted particles will be also analyzed from the perspective of fluctuation in processing parameters.

## Materials and methods

A compositionally graded Ti-xMo (0 ≤ x ≤ 12 wt %) specimen was produced using an Optomec LENS™ 750 at the University of North Texas from high purity elemental metal powders of Ti (99.9% pure, − 150 mesh from Alfa Aesar) and Mo (99.8% pure, − 100 + 325 mesh from Micron Metals). In this first AM system, the laser is a fixed optic Nd:YAG laser operating at 1064 nm provided by US Laser. The laser was operated between 350 and 500 W, and the atmosphere was kept elow 20 ppm oxygen. In addition, a series of Ti-15 wt% Mo alloys were deposited using an Optomec LENS™ system at Ames Laboratory. In this second AM system, the laser is a fiber optic Nd:YAG laser operating at 1064 nm provided by IPG. The IPG laser was operated between 183 and 367 W, and the atmosphere was kept below 5 ppm oxygen.

In both of these LENS™ systems, a computer-aided design (e.g. CAD) file is used in LENS™, from which a tool path is extracted for the subsequent laser deposition of a three dimensional specimen. The CAD file is converted and sliced into layers with a nominal thickness of 0.25 mm. Each layer consists of multiple parallel lines with a nominal hatch width of ~ 0.38 mm. The tool path that is generated based upon these variables is used to control the motorized stages (*x, y*) and a deposition head consist of focusing lens and powder nozzles mounted on the *z* motorized stage. The 2D (x, y) in-plane motion of the stage accompanied by—z vertical motion of the deposition head produce near-net-shape metallic pieces.

This research conducted two separate aspects. The first research activity used the fixed-optic laser equipped with two powder feeders to assess the influence of Mo on the microstructure, in particular on the grain size and texture. To conduct this first research activity, the LENS™ is equipped with two independently controlled powder feeders, which were loaded with pure Ti powder in powder feeder #1, and with a Ti-12 wt% Mo mechanically mixed elemental powder blend in powder feeder #2. An inert gas (here Ar) carries the powders from powder feeders into a controlled atmosphere box. The fluidized powders are injected (by four convergent Cu nozzles) into a localized melt pool created by a focused high energy Nd:YAG laser and an energy density of 10.2 MJ in^−3^ (~ 622.4 kJ cm^−3^). A 6 mm thick Ti-6Al-4V substrate was used as the base for the laser deposition of the powder blend and in situ alloying. The dimensions of the deposited rectilinear graded specimen was 38 mm × 25 mm × 12 mm rectilinear solid. The independent computer control of the powder flow rate allows for pre-programmed incremental changes in the relative mass flow rate from powder feeders and consequently variation in the local composition along the length of the sample.

The second research activity used the fiber-optic laser to assess the influence of processing parameters on the microstructure evolutions. Specifically, in this second study the travel speed and power were changed between ~ 8.5 and ~ 12.5 mm/s and between 183 and 367 W respectively, to vary the energy density between 2.4 MJ in^−3^ (~ 149 kJ/cm^−3^) and 7.4 MJ in^−3^ (~ 448 kJ cm^−3^) [31]. Nine depositions were made within the range of energy densities. The geometry of these deposits are right cylinders with a diameter of 7.62 mm and a height of 12.7 mm.

Following depositions, the deposits were sectioned from the substrate and cut in half longitudinally (*z* deposition direction). The specimen cross section was then prepared for materials characterization using conventional metallographic techniques. The sections were ground using 240–800 grit wet/dry SiC abrasive papers followed by polishing using a 0.04 colloidal silica suspension. Following preparation, the specimen was cleaned using a solution sequence of acetone, water–surfactant mixture, water, and methanol. Imaging of the microstructure was carried out using a FEI™ Quanta 250 FE-SEM equipped with a field emission gun (FEG) source and a backscatter detector and an Lx-31’s Optical Microscope. The local compositions along the graded specimen were determined using standardless energy dispersive spectroscopy (EDS) and the values are reported to the nearest whole wt.%. Average grain size measurements following the intercept method [32] were conducted for further analysis and interpretation.

## Results and discussion

### Composition effect on the as-deposited microstructure

**Fig. 1:**
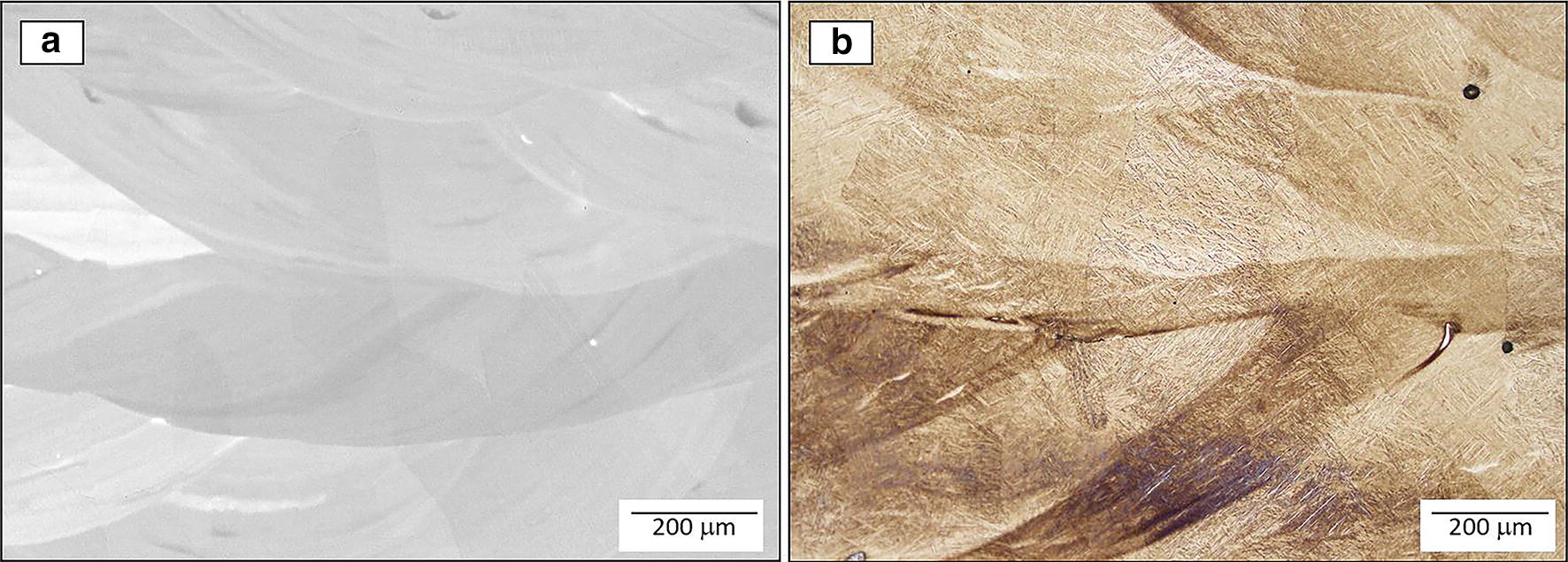
Micrographs corresponding to the compositionally graded Ti-xMo (0 ≤ x ≤ 12 wt %) specimen **a** backscatter SEM top, **b** optical micrograph bottom

Macrostructure observations revealed that the compositional gradient is columnar in nature, with the axis of growth in the build direction of the deposit (Fig. 1b). The expected columnar morphology of the prior-beta grains in additive manufactured specimens has been widely reported in several studies [12, 15, 33]. The prior-beta grain growth is preferential in the direction with the highest thermal gradient. In additive manufacturing, the z-direction (build direction) generally offers the highest thermal gradient because the heat source and the heat sink (substrate) are well positioned for that condition [33], although it can be disrupted under certain conditions [34, 35]. However, as observed in Fig. 1a, b the columnar grains are not perfectly aligned in the z-direction, yet still obey the principal thermal gradient, as they are perpendicular to the bottom of the molten pool. It can be attributed to a slightly tilting associated with the direction of laser motion [12] or due to the Marangoni effect in the molten pool that is generating a local highest thermal gradient in the perpendicular direction of the bottom in the molten pool [29]. In addition, Fig. 1a, b shows a discontinuity that is often termed as a “fish-scale” pattern [36, 37]. This feature could be a consequence of macrosegregation [6] or an inherent result of the layer by layer deposition in additive manufacturing [38].

**Fig. 2:**
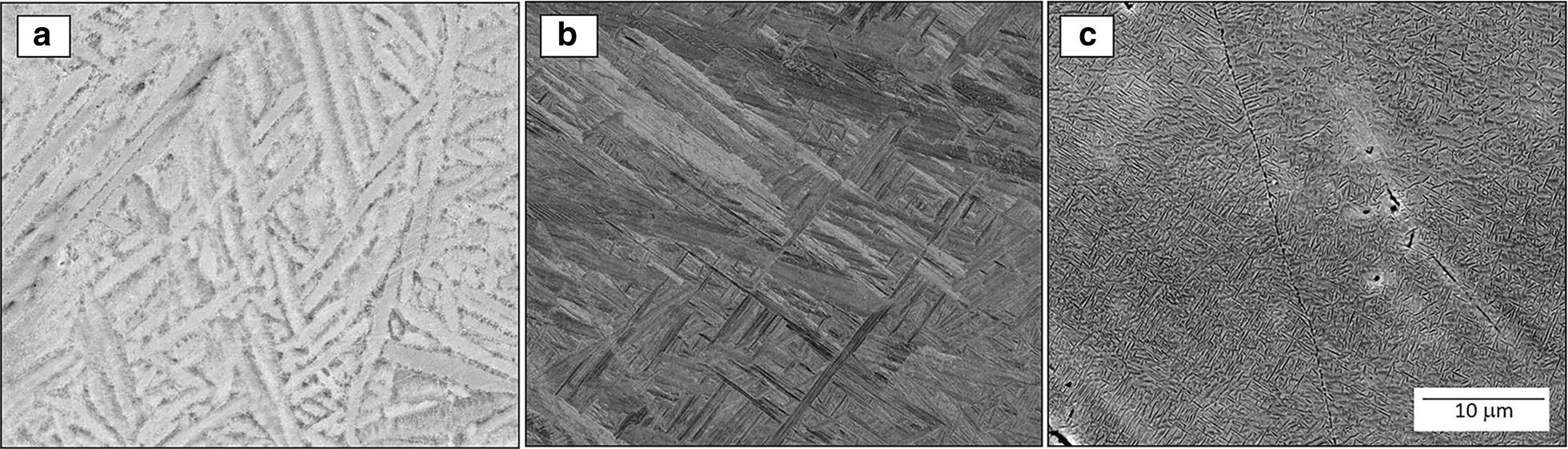
Backscatter SEM micrographs corresponding to the local average composition of **a** Ti-0.9 wt.% Mo, **b** Ti-2.8 wt.% Mo, **c** Ti-8.2wt.% Mo

Backscattered electron micrographs shown in Fig. 2a–c were taken along the graded specimen from the regions with a local average composition of 0.9 wt.%, 2.8 wt.% and 8.2 wt.% of Mo, respectively. For the entire gradient, the morphology is columnar beta grains with alpha laths within them. There is no evidence of reduction in the prior-beta grain width because of Mo additions across the gradient. To explain these results in terms of grain refinement mechanisms that are *inoperable* in these specimens, two different scenarios were reported in the Easton & St. John model as the possible mechanism of grain refinement in Ti-alloys by addition of an alloying element [16, 17, 39]. The first is the solute-based mechanism which is expressed and includes are growth restriction factor *Q* [40], while the second is a nucleant-based mechanism. In this case, molybdenum has a low *Q* factor of about 6.5*C*_*0*_ [17] which indicates that at least from the perspective of solute-based mechanism, Mo is not a good grain refiner. The low presence of unmelted particles across the Ti-xMo gradient and the columnar morphology of the grains from bottom to top indicate that the nucleant-based mechanism is not operating. This is in contrast to the previous results of Ti–W system [29], where both refinement mechanisms were responsible for grain refinement. In that case, tungsten was acting as a good grain refiner due to its high *Q* factor of about 22.65C_0_ and unmelted W particles were acting as a good source of new nuclei, possibly due to the high energy required to melt tungsten (~ 120 kJ mol^−1^) compared to the energy required to melt molybdenum from room temperature (~ 97 kJ mol^−1^).

Microstructure observations of Fig. 2a–c revealed that the size and aspect ratio of the alpha laths are being reduced inasmuch the molybdenum content is increasing. Figure 2c shows very small alpha precipitates and a visible increment in the beta fraction at 8.2 wt.% Mo. This variation in microstructural features with the addition of Mo is consistent with the literature [28]. However, it is very important to point out that in an individual specimen with no variation in composition, the alpha laths tend to be bigger at the top and smaller at the bottom as reported by Wu et al. [41], most likely due to variations in the heat transfer and cooling rates. The substrate acts as a heat sink, producing faster cooling rates at the bottom compared to the top. It is well known that faster cooling rates correspond with smaller the grain (or here, precipitate) sizes. Based on this analysis and the results, the alpha laths variation observed here can be attributed to the compositional effects.

### The effect of energy density on the as-deposited microstructure

**Fig. 3:**
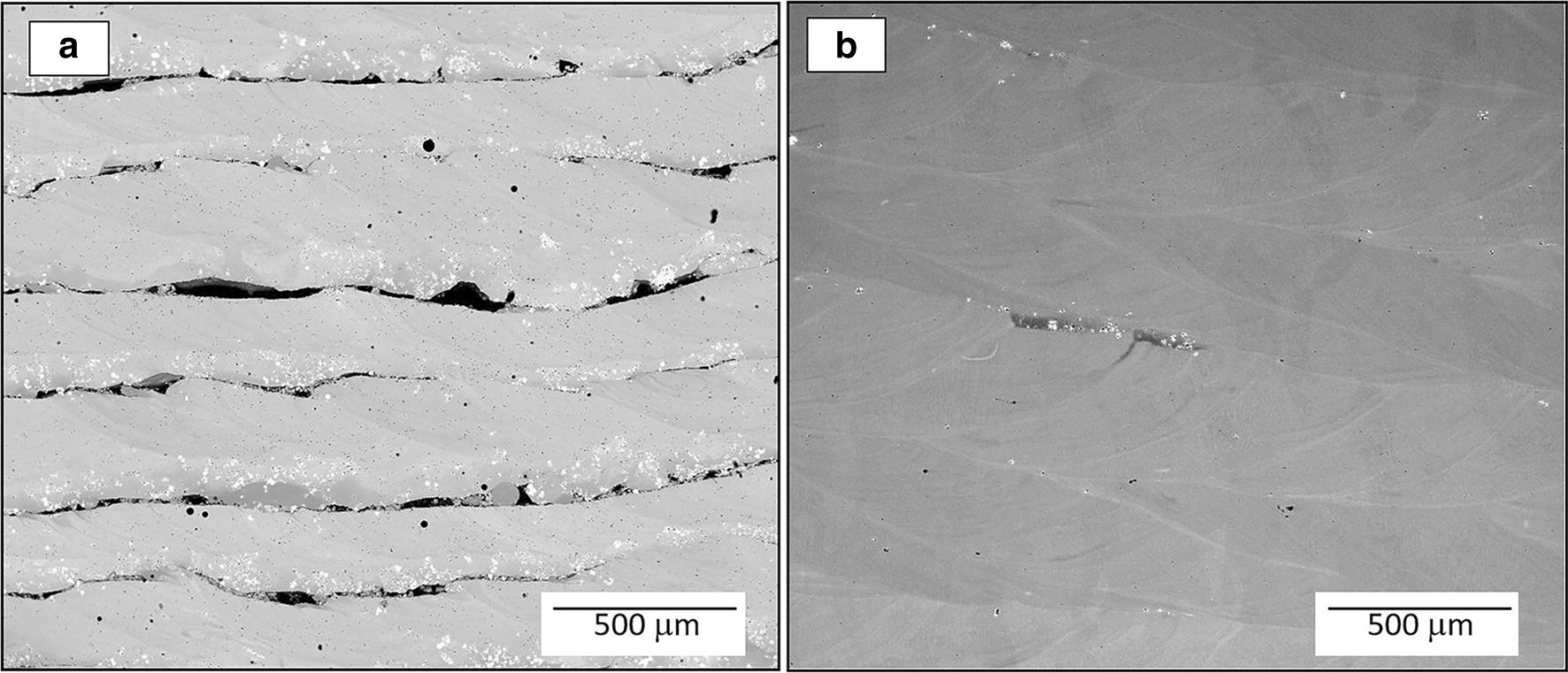
Backscatter SEM micrographs for two deposited specimens **a** 2.4 MJ in^−3^ (~ 149 kJ cm^−3^) and **b** 7.4 MJ in^−3^ (~ 448 kJ cm^−3^)

**Fig. 4:**
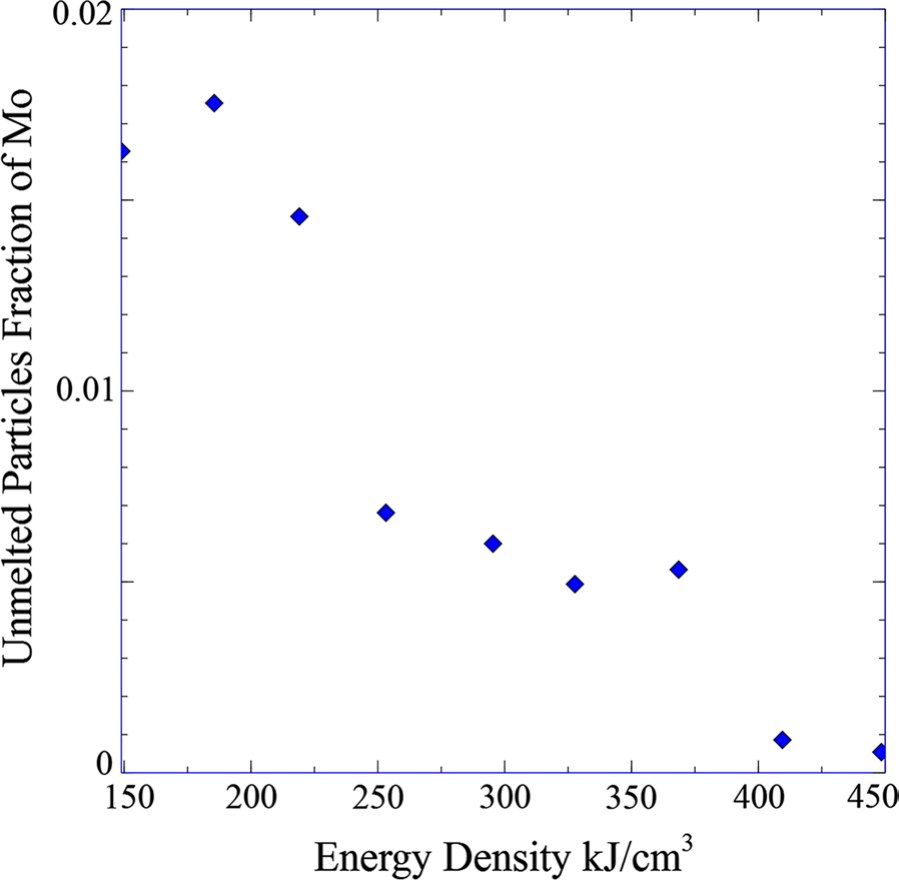
Fraction of unmelted particles of Mo as a function of energy density

Backscattered electron micrographs showing large fields in the *xz* plane for the highest and lowest energy density deposited specimens (Ti-15Mo fixed composition) are presented in Fig. 3a, b for 2.4 MJ in^−3^ (~ 149 kJ cm^−3^) and 7.4 MJ in^−3^ (~ 448 kJ cm^−3^), respectively. As expected, the specimen with the highest energy density shows more compositional homogeneity and so much less unmelted particles fraction. On the other hand, the specimen with the lowest energy density contains multiple lack of fusion regions between the deposition layers, some of them as large as 500 μm in length. The relationship between the unmelted particles fraction and the energy density across the nine specimens with different energy densities are shown in Fig. 4. The trend in this relationship is similar to the Ti–W system. However, at higher energy densities the fraction of unmelted particle for the Ti-Mo system is smaller than the corresponding value for Ti–W system. Therefore, when comparing the Ti-Mo results here, they require far less energy than the Ti–W binary system [29, 42]. This observation is also an indication of the reason for the ineffective nucleant-based mechanism of grain refinement discussed in the previous section for the gradient specimen.

Despite the fact that the set of specimens were deposited at a fixed composition of Ti-15Mo, it is clear that a compositional fluctuation exists over large length scales. The average atomic mass strongly influences the contrast in the backscattered electron micrographs as shown in Fig. 3a, b. Consequently, the brighter regions correspond to Mo rich regions and the darker gray regions correspond to Ti rich regions. Similar to the Ti–W system [29], in Fig. 3b the darker (Ti-rich) regions are near of Mo particle cluster due to the additional energy required to melt the Mo particles. In addition, the borders of the molten pools (fish scale patterns) are brighter zones corresponding to higher Mo content that will have an effect on the alpha laths precipitation. Molybdenum is a β-stabilizer element in titanium alloys.

**Fig. 5:**
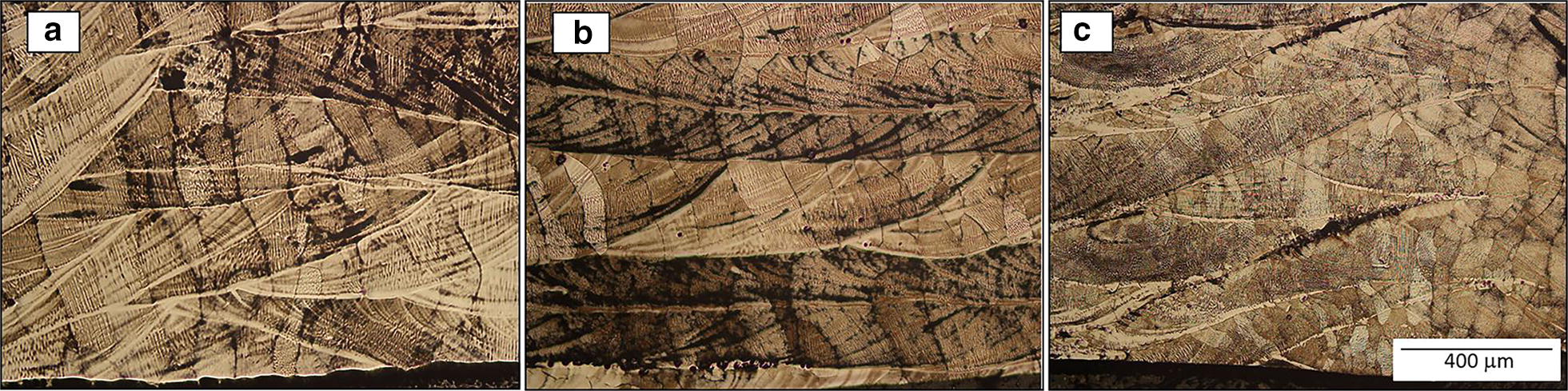
Optical micrographs at equivalent locations corresponding to different energy density specimens **a** 7.4 MJ in^−3^ (~ 448 kJ cm^−3^), **b** 6.04 MJ in^−3^ (~ 369 kJ cm^−3^) **c** 4.15 MJ in^−3^ (~ 253 kJ/cm^−3^)

**Fig. 6:**
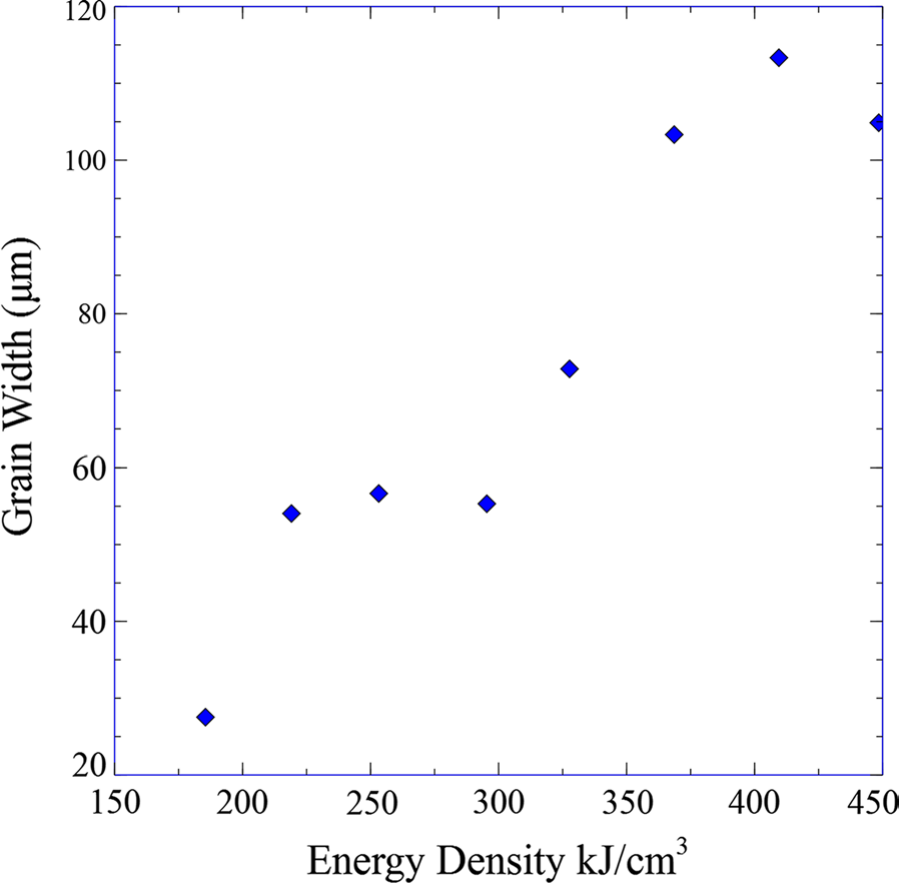
Grain size as a function of energy density

Figure 5a–c shows optical macro-morphologies of the Ti-15Mo specimens at the same location with three different energy densities.^1^ The macrostructures of all of the deposits were columnar in nature. The energy density term has been associated to properties and defects in previous studies [34, 43] and here it is related to the resultant columnar grain size (width). Figure 6 shows the variation of grain width with energy density. Inasmuch as the energy density increases, the columnar grain size (width) also increases, identical to what was observed previously for the Ti-xW system. Higher energy densities lead to bigger molten pools that translates to slower cooling rates. Therefore, there is a clear relationship between grain size and cooling rate, which is expected.

## Conclusions

A compositionally graded Ti-xMo specimen (0 ≤ x ≤ 12 wt %) was produced to determine the applicability of the Easton & St. John model concepts to the grain refinement effect of molybdenum and the associated mechanisms in Ti-based alloys. No reduction on the prior-beta grain size was observed and the morphology was columnar in nature across the entire gradient. The analysis of these results from the perspective of the two possible refinement mechanisms confirms their applicability. The low growth restriction factor for the Ti-Mo system of *Q *= 6,5C_0_ explains the unsuccessful refinement of the solute-based mechanism, but also importantly provides a guiding lower bound for the applicability of such growth restriction factors for additively manufactured titanium alloys. On the other hand, the low presence of unmelted particles in the specimen and the comparatively low energy required to melt Mo could explain the unsuccessful refinement of the nuclei-based mechanism.

The energy density and the grain size are proportional according to the results from the nine fixed composition specimens at different energy densities. More energy absorption from the material represents bigger molten pools, which in turn implicates lower cooling rates.

## Abbreviations

LENS™: Laser Engineered Net Shaping
AM: additive manufacturing
CAD: computer-aided design
EDS: energy dispersive spectroscopy

## Footnotes

1 Energy density may be defined in the following way:
$$\rho_{energy} = \frac{P}{{v \cdot t_{layer\;spacing} \cdot t_{hatch\;width} }}$$
where *P* is the laser power, *v* is the velocity of the laser, *t*_layer spacing_ is the layer spacing, and *t*_hatch width_ is the distance between passes. Both layer spacing and hatch width provide information regarding the radial distribution of temperature.
